# Clinical Outcomes of CAD-CAM Subperiosteal Implants for the Rehabilitation of Atrophic Jaws

**DOI:** 10.3390/dj12080241

**Published:** 2024-07-29

**Authors:** Giulio Gasparini, Mattia Todaro, Paolo De Angelis, Roberto Boniello, Gianmarco Saponaro, Edoardo Rella, Enrico Foresta, Horia Vasile Hreniuc, Francesca Azzuni, Ermal Pashaj, Alessandro Moro, Giuseppe D’Amato

**Affiliations:** 1 Maxillo-Facial Surgery Unit, IRCSS “A. Gemelli” Foundation—Catholic University of the Sacred Heart, 00168 Rome, Italy; giulio.gasparini@unicatt.it (G.G.); mattiatodaro@gmail.com (M.T.); gianmarco.saponaro@gmail.com (G.S.); enrico.foresta@policlinicogemelli.it (E.F.); francescaazzuni@gmail.com (F.A.); alessandro.moro@policlinicogemelli.it (A.M.); 2 Department of Head and Neck, Oral Surgery and Implantology Unit, Institute of Clinical Dentistry, Catholic University of the Sacred Heart, Polyclinic Foundation, 00168 Rome, Italy; dr.paolodeangelis@gmail.com (P.D.A.); roberto.boniello@unicatt.it (R.B.); 3 Department of Emergency, Intensive care Medicine and Anesthesia, Fondazione Policlinico Universitario A. Gemelli IRCCS, Largo Francesco Vito N 8, 00168 Rome, Italy; horiavasile.hreniuc@policlinicogemelli.it; 4 Maxillo-Facial Surgery Unit, Hospital Center Catholic University “Our Lady of Good Counsel”, 1000 Tirana, Albania; e.pashaj@unizkm.al; 5 Faculty of Medicine and Surgery, Unicamillus International Medical University, 00131 Rome, Italy; dottgdamato@gmail.com

**Keywords:** subperiosteal, implant, atrophic, edentulous, jaw

## Abstract

Thanks to the use of new digital technologies and innovations in materials, there has been an increasing interest in subperiosteal implants. These implants are considered to be helpful for the rehabilitation of severe jaw atrophies, as they overcome some disadvantages of endosseous implantology. In the present clinical retrospective study, 18 patients were treated and the clinical outcomes of the treatment were recorded after 12 months of follow-up.

## 1. Introduction

Nowadays, the rehabilitation of atrophic jaws with conventional endosseous implants is the gold standard for fixed rehabilitations; nevertheless, the treatment of severely atrophic jaws still represents a challenge. 

Several bone-grafting techniques have been described in the literature, from guided bone regeneration to onlay/inlay grafts, the Khoury technique, and alveolar ridge splitting. However, these options may be used only in selected patients and have a high risk of complication and failure. Furthermore, specific technical skills are required and several surgeries may be needed [1].

Alternatives to bone grafting procedures, according to the clinical scenario, are represented by short implants, narrow implants, and tilted implants [2]. In case of an edentulous patient affected by severe atrophy, pterygoid and zygomatic implants may be used for immediate rehabilitation. 

Another option is provided by subperiosteal implants. These implants, introduced in the 1940s, were once widely used to treat edentulous maxillary and mandibular arches, particularly in cases of severe bone atrophy. Subperiosteal implants are beneficial for patients with significant alveolar arch resorption because, unlike endosseous implants that are embedded deeply within the bone, they provide a framework that sits on top of the maxilla or mandible beneath the periosteum. However, their popularity waned over time due to problems such as the complexity of impression procedures, high infection rates, and difficulties with implant positioning. This decline led to a preference for endosteal implants, influenced by Dr. Brånemark’s groundbreaking work on osseointegration.

In recent years, the literature has shown an increasing interest in custom-made subperiosteal implants for the rehabilitation of severe atrophy of the jaws, thanks to the advent of 3D CAD-CAM technologies and innovations in materials [3,4,5]. 

Furthermore, advancements in manufacturing technologies, along with the use of materials like titanium or titanium alloys, have significantly enhanced the quality of these implants [6]. This has led to better precision, fit, and durability of subperiosteal implants, making them a reliable and efficient option for patients with atrophic arches.

The subperiosteal implant is a titanium grid applied to the outer surface of the bone and fixed with screws; the structure has specifically designed abutments to support the dental prostheses.

Advantages of this technique include the possibility of avoiding bone grafting and related donor-site morbidity, reducing both the number of surgeries and their invasiveness, and the possibility of performing an immediate loading protocol.

The purpose of this retrospective study is to analyze the clinical outcomes of fully edentulous patients rehabilitated using subperiosteal implants, alongside a digital workflow.

## 2. Materials and Methods

This retrospective clinical study was based on the clinical records of patients treated in the department of Oral and Maxillofacial Surgery of the Catholic University of the Sacred Heart of Rome from January 2019 to December 2021.

The study involved fully edentulous patients treated with subperiosteal implants with complete pre- and postoperative clinical and radiographic documentation, having one year of follow-up.

This retrospective study was conducted in accordance with the Helsinki Declaration on Human Subject Experimentation. Because of the retrospective nature of the study, an exemption was granted by the local ethics committee.

This retrospective study was assessed by the authors to be very low-risk; thus, ethics approval was not required.

A total of 18 patients who had been treated with subperiosteal implants were enrolled in this study. 

Among the 18 patients, 10 were females and 8 were males aged between 40 and 80 years, with a mean age of 62.39 ± 11.07 years. 

Patients with contraindications to implant surgery were excluded from the analysis, such as those with uncontrolled diabetes, immunocompromised patients, those who had been treated with bisphosphonates, and those who had undergone previous radiation therapy.

Patients treated with conventional implant surgery or with bone augmentation procedures having incomplete clinical records were also excluded. 

Regarding the manufacturing of subperiosteal implants, the work involved six different companies, all based in Italy and with a proven experience of at least five years in the dental and maxillofacial field.

All of the implants were made of titanium with screw-retained connections, nine of them were maxillary and nine mandibular, and 13 cases were full-arch rehabilitation. 

The occurrence of intraoperative and postoperative complications was recorded. The implant survival and success rates were investigated as well as patients’ satisfaction. An implant was defined as having survived if it was still in use at the end of the follow-up, while it was defined as a success if it encountered no complications up to the end of the follow-up. The questionnaire was administered to all of the patients before and at least one year after the prosthesis delivery. It consisted of four questions about aesthetic satisfaction, chewing ability, phonetics, and willingness to repeat the treatment.

The score for each question ranged from 0 to 10, with 0 representing the worst possible outcome and 10 the best possible outcome. 

### 2.1. Workflow

The clinical protocol used in the present study to rehabilitate the edentulous arch with subperiosteal implants involved different stages: data collection, design and implant construction, surgery, and prosthesis delivery.

**Figure 1:**
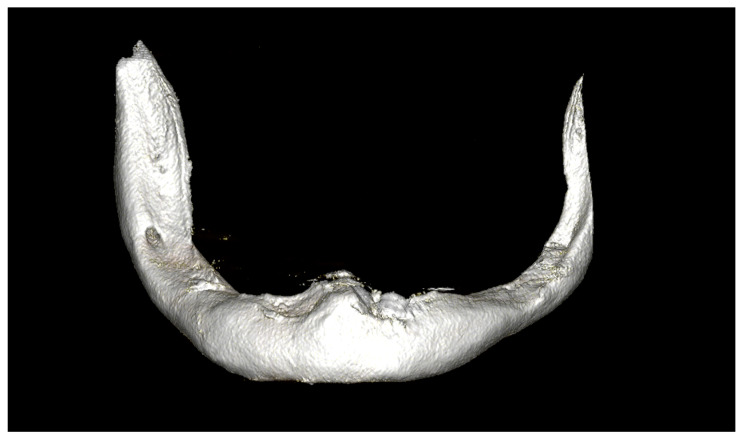
CBCT scan of severely atrophic lower jaw.

In the data collection phase, every patient underwent orthopantomography, cone-beam computed tomography (CBCT), dental impression taking (Figure 1), interocclusal relationship recording, and clinical evaluation. 

In this phase, the following patients were excluded: those with an active infection process, uncontrolled diabetes, immunosuppression, treatment with bisphosphonates, and previous radiation therapy.

If the patient already had a mobile prosthesis, it was used as a diagnostic wax-up; otherwise, one was created. 

**Figure 2:**
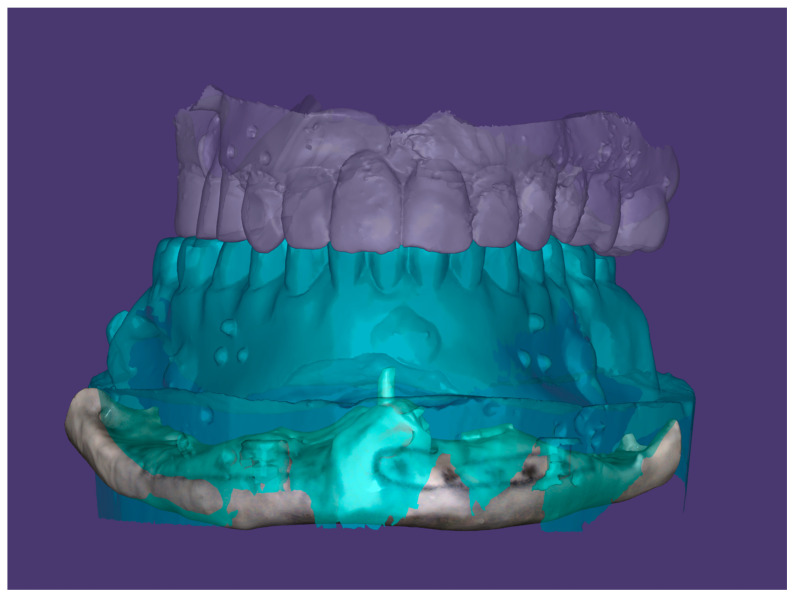
First phase of digital design process: radiological template, opposing arch, and edentulous jaw are acquired.

A radiological template, required to virtually plan the position of the implants, was prepared from the diagnostic wax-up and worn by the patient during the execution of CBCT; after that, the radiological template alone was scanned (Figure 2).

Finally, the patients’ intraoral scans were saved as STL files and used to evaluate the anatomy and height of the mucosa, as well as to define the correct height of the prosthetic abutments. Intraoral scans were needed to properly position the abutments and to properly plan the surgery and the extent, if needed, of the osteotomy.

When possible, a 3D-printed stereolithographic model of the atrophic jaw was produced.

**Figure 3:**
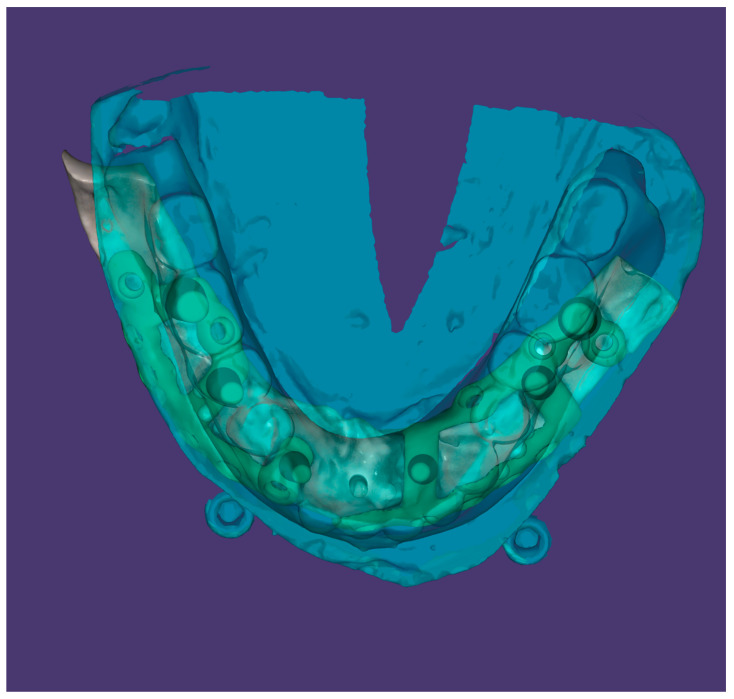
Digital design of the subperiosteal implants, the diagnostic wax-up guide, and the positioning of the prosthetic posts.

The digital implant positioning was based on the prosthetic plan and on the occlusion, which also determined the positioning of the prosthetic abutments (Figure 3).

The retaining arms of the implant were designed to reach areas of bone with adequate characteristics to avoid the emergence of structures such as the mental and infraorbital nerves. Usually, the retaining arms in the maxilla are planned where the basal bone provides optimal thickness for screw positioning. In this phase, the holes to retain the implant were planned, taking into consideration the screw direction and length in order to avoid structures such as the maxillary sinus and considering a screw direction that could allow for proper placement during the surgery.

Alveolar ridge ostectomies were planned below every post. These bone reduction areas were thought to avoid or minimize implant protrusion from the bone profile of the alveolar crest, reducing the risk of implant exposure.

In cases of insufficient bone height at the level of the maxillary sinus floor or at the level of the inferior alveolar nerve, the ridge ostectomy was not planned; however, the placement of abutments was avoided in sites that did not permit ridge ostectomies whenever possible.

**Figure 4:**
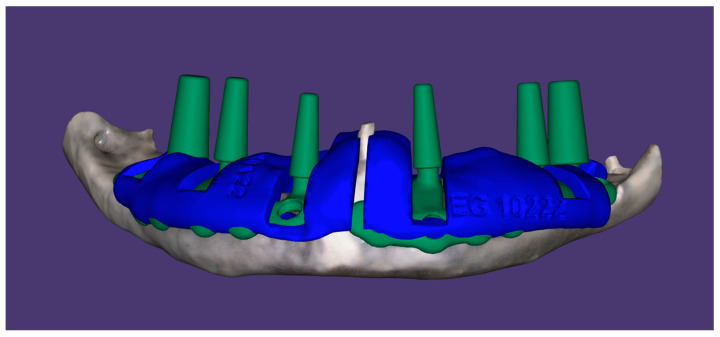
Digital design of the subperiosteal implants; the guides for bone ostectomies are planned.

A surgical guide for the ostectomies was designed in order to direct the ostectomy slots and to modify the alveolar crest profile, if needed (Figure 4).

An initial resin model of the implant was produced for verification, and after its approval, the final implant was created. 

The implant was manufactured by selective laser melting. 

The surgery was conducted under local anesthesia, or under local anesthesia with sedation in case of bilateral implant positioning. A full-thickness mucosal incision was conducted along the edentulous ridge and a subperiosteal flap elevation was performed. 

Bone reduction ostectomies were then performed with a round bur after positioning the surgical guide.

**Figure 5:**
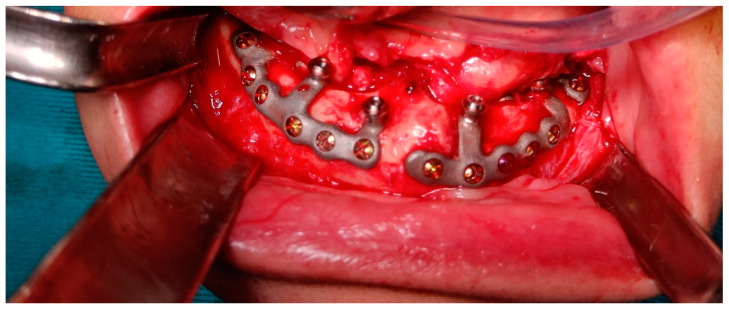
Subperiosteal implant positioned after rigid fixation.

The implant was then positioned, and the fixation screws were applied after a meticulous fitting check between the implant and the bone surface (Figure 5).

**Figure 6:**
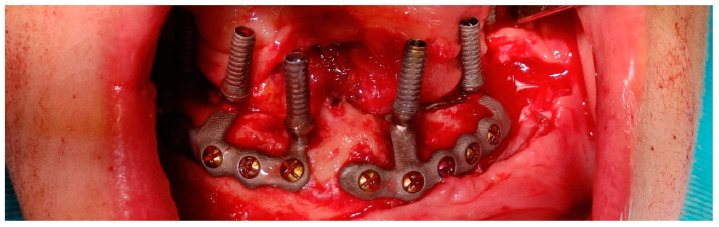
Temporary abutment connections screwed over the abutments before fitting the temporary prosthesis.

Temporary abutment connections were screwed over the abutments (Figure 6) and a temporary prosthesis was positioned in proper occlusion with the opposite dental arch. The provisional prosthetic denture was fixed to the temporary abutment connections with acrylic resin, obtaining immediate loading. 

**Figure 7:**
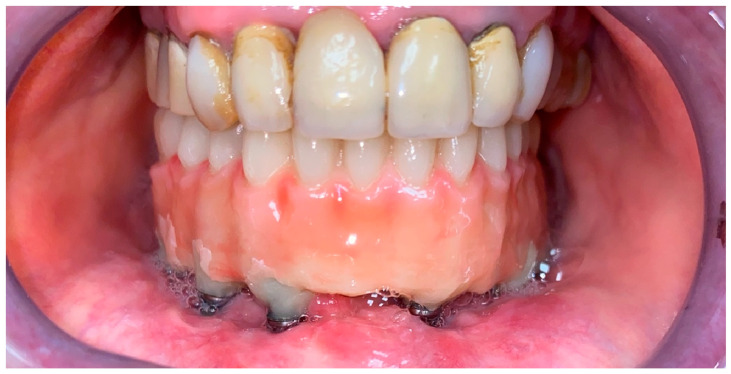
Postoperative control one month after surgery showing proper soft-tissue healing.

The temporary prosthesis was then finished, taking care to eliminate any area of possible compression of the wound or mucosa, in order to create a surface free of roughness and thus cleanable (Figure 7).

During the postoperative phase, the patient was instructed to follow a proper hygiene protocol and a semiliquid diet. In addition, oral antibiotics were prescribed, in particular amoxicillin plus clavulanic acid, 1 g every twelve hours for six days, and Chlorhexidine mouthwash every eight hours until suture removal.

The definitive prosthesis was delivered 3–5 months after the complete healing.

### 2.2. Statistical Analysis

Qualitative variables were described as absolute and percentage frequencies, while quantitative variables were summarized as mean, median, minimum, maximum, and standard deviation. The Shapiro–Wilk test was used to check for normality, keeping a statistical threshold set at *p* < 0.05, and given the obtained results, a parametric test (*t*-test for paired results) was adopted to compare the questionnaire results obtained at baseline (T0) with the results obtained at the last follow-up (T1).

## 3. Results

All the patients were followed up for one year after receiving their final prosthesis. 

The implant survival rate was 100%. No postoperative mobility of the implants was observed at any time. 

Three intraoperative complications were recorded: a case of a poorly finished implant with the presence of imperfections in the screw retaining hole, which caused incomplete screwing; a case with non-properly angulated and non-parallel prosthetic abutments; and a case in which the prosthetic abutment protruded too much from the mucosa, making the prosthetic fitting difficult.

Among the postoperative complications, only one patient showed a minimal mucosal exposure of the implant with no signs of infection three months postoperatively, to date without functional consequences. The implant success rate was 94.44%.

The post-treatment questionnaire administered one year after the prosthesis delivery revealed a significant increase in relation to all four parameters investigated (Table 1). The aesthetic satisfaction at the baseline was 3.06 ± 1.51 and 5.61 ± 0.98 at the follow-up (*p* < 0.001); the chewing ability increased from 3.94 ± 1.55 to 6.89 ± 0.90 (*p* < 0.001), and the phonetics from 5.33 ± 0.91 to 6.44 ± 0.62 (*p* < 0.01). The comfort of these patients went from 4.28 ± 0.89 at the baseline to 6.83 ± 0.86 at the follow-up (*p* < 0.001). 

## 4. Discussion

Subperiosteal implants were described in the 1940s [7]. However, they were gradually abandoned in favor of endosseous implants due to their complications, such as implant exposure, mobility, and failure. 

Moreover, before the advent of 3D technologies, an initial surgery was needed to obtain the impression of the atrophic jaw and to create the implant, and a second surgery to position the implant [8]. The main problems reported in relation to subperiosteal implants were the following: material fracture, implant mobility, lack of osseointegration, peri-implantitis, and implant exposure [9,10,11,12]. Bone resorption is a well-known complication of subperiosteal implants. In this regard, the literature indicates a higher failure rate in maxillary subperiosteal implants compared to mandibular subperiosteal implants [10].

The issues encountered in the past were related to the lack of precision, inadequate material, and the poor design of the implant.

In the past, the use of Vitallium, which has a higher Young’s modulus compared to the cortical bone, caused the phenomenon of stress shielding with resorption of the underlying bone. Moreover, Vitallium has no soft-tissue or bone integration properties. 

Nowadays, the use of titanium rectifies this problem and allows the osseointegration of the implant [13].

As described by McAllister [14], in the 1990s, advances in computed tomography and the advent of stereolithography were applied to the fabrication of subperiosteal implants. In 2018, Cerea and Dolcini shared their experience with 70 patients treated with custom-made direct metal laser sintering (DMLS) titanium subperiosteal implants with a survival rate of 95.8% over a two-year follow-up period [10].

Mommaerts et al. described an innovative implant design that allowed the disconnection of any post from the basal loop in case of the development of peri-implantitis. They also pre-planned the screw length to avoid piercing the Schneiderian membrane so that maxillary sinus disease could be averted [15]. As described by Negrini et al., subperiosteal implants are also a valid solution in cases which present reduced prosthetic spaces and reduced bone vertical dimension [16]. According to our experience, subperiosteal implants represent a valid tool for the rehabilitation of severe jaw atrophies.

In our series of patients, no major complications were encountered, obtaining good rehabilitation in all cases. Nevertheless, many issues were experienced. The first problem encountered was related to communication with the company, in particular to the difficulty in communicating with the bioengineer, which resulted in long waits between the various modifications. This resulted in prolonged manufacturing times and surgery delays. We believe that the first design phase should result from a close collaboration between the clinician and the bioengineer. As described by Obwegeser years ago, the cooperation between the surgeon and the prosthodontist is of paramount importance in planning the implant [17]. This is unquestionable, especially regarding the choice of the number and position of the prosthetic posts.

Another issue was linked to the material preparation and delivery, since the companies involved had different methods of sterilization and delivery of the implantable material. In our opinion, the implant should be provided packaged in a double bag after electrical discharge machine polishing is performed, and the decontamination and sterilization processes should be clearly specified and dated.

Also, from our point of view, a stereolithographic model is very useful to plan subperiosteal implants; therefore, this device should always be supplied by the manufacturers.

Protective caps for screw connections (MUAs) are needed to avoid inadvertent damage to the components, which could impair the positioning of a screw-retained prosthesis.

The screws used for the implant positioning should ideally be provided by the company, with a dedicated screwdriver.

It is advisable that together with the final implant, a stereolithographic model of the edentulous jaw and a copy model of the implant are provided.

Although many authors describe the use of subperiosteal implants without bone resection areas, we believe that this method allows for maintenance of the profile of the implant at the basal bone height, therefore below the alveolar bone, which is subject to resorption over time. Moreover, it allows for reduction of the prominence of the implant from the bone profile, thus decreasing the risk of mucosal exposure. As described by Negrini et al., bone ostectomies are also needed in the presence of undercuts that may prevent the fitting of the implant [16].

One more problem encountered was deficient design of surgical templates for bone reduction areas. Companies often design templates with scarce consideration of soft tissues and surgical approaches, thus leading to difficulties in the guide positioning during the surgery. Furthermore, the cutting guides are usually printed in resin and not in titanium, leading to two main issues: first of all, the guide is more flexible, causing possible imprecision; secondly, during the drilling of the bone, the guide can be easily damaged with consequent imprecision and powder dispersion in the operating field.

Rinaldi et al. described the use of titanium alloy resection guides for subperiosteal implants [18].

In our opinion, a metallic guide is the best possible option. In case of poor fitting, a flexible cutting guide can be adapted to the bone, leading to an imprecise positioning of the implant. Any imprecision will also result in prolonged surgical time and more need for implant manipulation with an increased risk of contamination.

In one case, poorly finished implants with the presence of imperfections in the holes for the retaining screws were found, resulting in incomplete descent of the screw head. All other intraoperative complications were related to the prosthetic rehabilitation of these implants; we further advise clinicians who want to use this procedure to be extremely precise in planning their surgery, as these complications can be quite cumbersome and time-consuming. No surgical complications were observed, highlighting the safety of this surgery. Regarding the only postoperative complication, which is exposure of the implant, this is mainly related to a lack of proper oral hygiene and to a reduced thickness of soft tissues; this complication should be avoided, and a first-intention healing at the time of implant placement is of great help.

When the rehabilitation of a dental arch is performed with two separate implants, the use of a connection bar is advisable to allow a precise fitting. The connection bar, applied during the fixation of the implants, guarantees precision in the positioning between the implants. A lack of precision in this procedure can lead to an altered position of the abutments with consequent difficulties during the prosthesis phase. Van den Borre et al. described the use of a connecting bar structure in case of double-structure subperiosteal implants [19].

In one of the above-mentioned complications, the prosthetic abutments were not properly angulated and not parallel. This improper angulation increased the complexity of the immediate restoration in the operating room. This was obviously a design error, because one of the benefits of subperiosteal implants is that they are preoperatively designed and, therefore, the common problems in standard implantology can be avoided.

In another complication, the prosthetic abutment was protruding too much from the mucosa, which caused the prosthetic fitting to be difficult. An incorrect design can lead to an incorrect exit of the abutment from the gingival plane. As previously mentioned, during the design phase, maximum attention must be given to the position and height of the prosthetic posts.

Our article has many faults. First and foremost, only a reduced sample could be obtained, given that subperiosteal implants are not the first choice for treating total edentulism. Also, our follow-up is not particularly long, and no comparison was made between this intervention and other alternatives. Furthermore, no radiographical evaluation was attempted, and so no data can be provided. Further research should investigate these topics.

## 5. Conclusions

According to the present study experience, subperiosteal implants represent a valid tool for the rehabilitation of severe jaw atrophies.

This technique appears to be particularly useful for the treatment of complex cases and allows the patient to avoid treatments with longer timelines, such as bone grafting procedures. It also allows for less invasiveness than other techniques like zygomatic or pterygoid implants [20,21,22].

Despite the growing and renewed interest in this technique, well-defined protocols are still lacking, representing an issue that we have been able to discover by working with various companies that have proposed slightly different solutions in every step.

Furthermore, there are few studies with long-term follow-ups, and the results will need to be further investigated.

## Figures and Tables

**Table 1:** Results of the questionnaire.

T0				T1			
Q1	Q2	Q3	Q4	Q1	Q2	Q3	Q4
3.94 ± 1.55	3.06 ± 1.51	5.33 ± 0.91	4.28 ± 0.89	6.89 ± 0.90	5.61 ± 0.98	6.44 ± 0.62	6.83 ± 0.86

## Data Availability

The data presented in this study are available on request from the corresponding author.

## References

[1] Wortmann D.E., Boven C.G., Schortinghuis J., Vissink A., Raghoebar G.M. (2019). Patients’ appreciation of pre-implant augmentation of the severely resorbed maxilla with calvarial or anterior iliac crest bone: A randomized controlled trial. Int. J. Implant Dent..

[2] Lee E.A., Prasad H., Lynch S. (2024). Sequential Human Histology Results of the Subperiosteal Minimally Invasive Aesthetic Ridge Augmentation Technique (SMART): A Chronologic Wound Healing Proof-of-Principle Study. Int. J. Periodontics Restor. Dent..

[3] Bai L., Zheng L., Ji P., Wan H., Zhou N., Liu R., Wang C. (2022). Additively Manufactured Lattice-like Subperiosteal Implants for Rehabilitation of the Severely Atrophic Ridge. ACS Biomater. Sci. Eng..

[4] Elsawy M.A., ELgamal M.E., Ahmed W.M., El-Daker M.A., Hegazy S.A. (2022). Polyetheretherketone subperiosteal implant retaining a maxillary fixed prosthesis: A case series. J. Prosthet. Dent..

[5] Mourad K.E., Altonbary G.Y., Emera R.M.K., Hegazy S.A.F. (2023). Polyetheretherketone computer-aided design and computer-aided manufacturing framework for All-on-Four mandibular full-arch prosthesis: 3 Years’ retrospective study of peri-implant soft tissue changes and ridge base relationship. J. Prosthodont..

[6] Arshad M., Khoramshahi N., Shirani G. (2023). Additively custom-made 3D-printed subperiosteal implants for the rehabilitation of the severely atrophic maxilla (a case report). Clin. Case Rep..

[7] Linkow L., Wagner J.R., Chanavaz M. (1998). Tripodal mandibular subperiosteal implant: Basic sciences, operational procedures, and clinical data. J. Oral Implantol..

[8] Bodine R.L., Yanase R.T., Bodine A. (1996). Forty years of experience with subperiosteal implant dentures in 41 edentulous patients. J. Prosthet. Dent..

[9] Moore D.J., Hansen P.A. (2004). A descriptive 18-year retrospective review of subperiosteal implants for patients with severely atrophied edentulous mandibles. J. Prosthet. Dent..

[10] Cerea M., Dolcini G.A. (2018). Custom-made direct metal laser sintering titanium subperiosteal implants: A retrospective clinical study on 70 patients. BioMed Res. Int..

[11] Nguyen T.M., Caruhel J.B., Khonsari R.H. (2018). A subperiosteal maxillary implant causing severe osteolysis. J. Stomatol. Oral Maxillofac. Surg..

[12] Claffey N., Bashara H., O’Reilly P., Polyzois I. (2015). Evaluation of new bone formation and osseointegration around subperiosteal titanium implants with histometry and nanoindentation. Int. J. Oral Maxillofac. Implants.

[13] Strappa E.M., Memè L., Cerea M., Roy M., Bambini F. (2022). Custom-made additively manufactured subperiosteal implant. Minerva Dent. Oral Sci..

[14] McAllister M.L. (1998). Application of stereolithography to subperiosteal implant manufacture. J. Oral Implantol..

[15] Mommaerts M.Y. (2018). Evolutionary steps in the design and biofunctionalization of the additively manufactured sub-periosteal jaw implant ‘AMSJI’ for the maxilla. Int. J. Oral Maxillofac. Surg..

[16] Stefano N., Lorenzo V. (2021). The Use of Digital Sub-Periosteal Implants in Severe Maxillary Atrophies Rehabilitation: A Case Report. J. Head Neck Spine Surg..

[17] Obwegeser H.L. (1959). Experiences with subperios-teal implants. Oral Surg. Oral Med. Oral Pathol..

[18] Rinaldi M., De Neef B., Loomans N.A., Mommaerts M.Y. (2020). Guidelines for the use of resection guides for subperiosteal maxillary implants in cases of terminal dentition—A novel approach. Ann. Maxillofac. Surg..

[19] Van den Borre C., Rinaldi M., De Neef B., Loomans N.A.J., Nout E., Van Doorne L., Naert I., Politis C., Schouten H., Klomp G. (2021). Patient- and clinician-reported outcomes for the additively manufactured sub-periosteal jaw implant (AMSJI) in the maxilla. Int. J. Oral Maxillofac. Surg..

[20] Tzerbos F., Bountaniotis F., Theologie-Lygidakis N., Fakitsas D., Fakitsas I. (2016). Complications of Zygomatic Implants: Our Clinical Experience with 4 Cases. Acta Stomatol. Croat..

[21] Dryer R.R., Conrad H.J. (2019). Displacement of a Dental Implant into the Pterygoid Fossa: A Clinical Report. J. Prosthodont..

[22] Łoginoff J., Majos A., Elgalal M. (2024). The Evolution of Custom Subperiosteal Implants for Treatment of Partial or Complete Edentulism in Patients with Severe Alveolar Ridge Atrophy. J. Clin. Med..

